# Nutrition Knowledge, Attitudes, and Lifestyle Practices That May Lead to Breast Cancer Risk Reduction among Female University Students in Lebanon

**DOI:** 10.3390/nu16071095

**Published:** 2024-04-08

**Authors:** Nour Deeb, Farah Naja, Lara Nasreddine, Samer Kharroubi, Nadine Darwiche, Nahla Hwalla

**Affiliations:** 1 Department of Nutrition and Food Sciences, Faculty of Agricultural and Food Sciences, American University of Beirut, P.O. Box 11-0236, Riad El Solh, Beirut 1107 2020, Lebanon; nmd30@mail.aub.edu (N.D.); ln10@aub.edu.lb (L.N.); sk157@aub.edu.lb (S.K.); 2 Department of Clinical Nutrition and Dietetics, College of Health Sciences, Research Institute of Medical & Health Sciences (RIMHS), University of Sharjah, Sharjah P.O. Box 27272, United Arab Emirates; fnaja@sharjah.ac.ae; 3 Faculty of Agricultural and Food Sciences, American University of Beirut, P.O. Box 11-0236, Riad El Solh, Beirut 1107 2020, Lebanon; 4 Department of Biochemistry and Molecular Genetics, Faculty of Medicine, American University of Beirut, P.O. Box 11-0236, Riad El Solh, Beirut 1107 2020, Lebanon; nd03@aub.edu.lb

**Keywords:** nutrition, knowledge, attitude, practices, breast cancer, female university students, breast cancer

## Abstract

Research has identified both nonmodifiable and modifiable risk factors for breast cancer (BC), with accumulating evidence showing that adopting adequate dietary practices could decrease the risk of this disease. This study aimed to assess nutrition knowledge, attitudes, and lifestyle practices (KAP) that may lead to BC risk reduction among female university students in Lebanon and examine the determinants of their practices. A cross-sectional survey was conducted using a convenience sampling method, comprising 356 (response rate: 71.2%) female students at the American University of Beirut aged 18 to 25 years with no history of BC. Participants completed a pre-tested questionnaire addressing the objectives of the study. The modified Bloom’s cut-off of 75% was used to categorize knowledge and practice scores as poor or good and attitudes as negative or positive. Large proportions of students had poor knowledge (68.3%), negative attitudes (65.4%), and poor practices (98.0%) scores. Pursuing a health-related major and having a higher GPA were associated with better knowledge and attitudes while being older and having a lower degree of stress were associated with positive attitudes only. Having a lower body mass index (BMI) was associated with better practice scores. Better knowledge significantly predicted higher intake of fruits and vegetables. Overall knowledge and attitudes were significantly correlated with each other, but neither was significantly correlated with overall practice. These findings underscore the importance of implementing public health programs geared towards improving nutrition KAP that may lead to BC risk reduction.

## 1. Introduction

Breast cancer (BC) is a significant public health concern, representing one in eight cancer diagnoses globally [1,2,3]. It is also the leading cause of cancer death among women worldwide, accounting for around 670,000 deaths (15.4% of cancer deaths) in 2022 [4,5,6]. In Lebanon, BC is the most commonly diagnosed cancer in women, where BC cases accounted for 16.6% of cancers in both sexes and 33.6% of cancers among females in 2022 [7]. The median age at BC diagnosis is around 53 years in Lebanon, which is below the ages of 63 and 68 years reported in the US and Europe, respectively [8,9,10]. Among the modifiable BC risk factors, the high prevalence of obesity and smoking in the Lebanese population were noted as possible contributing factors to this increased incidence [11,12]. 

While both nonmodifiable and modifiable risk factors have been identified for BC [13,14], evidence has demonstrated that only around 5 to 10% of BC cases can be attributed to inherited gene mutations, mainly mutations in *BRCA1* and *BRCA2* genes [15,16]. Other less common gene mutations have also been reported to increase the risk of BC, including *ATM*, *PALB2*, *TP53*, *CHEK2*, *PTEN*, *CDH1*, and *STK11*, among others. According to the “Nordic Twin Study of Cancer”, the heritability of liability to BC is around 31% [17]. On the other hand, 35% of cancer incidence worldwide can be linked to unhealthy dietary practices and lack of physical activity, highlighting the importance of addressing these factors, especially in younger female age groups [18]. 

Research findings have revealed that maintaining a healthy weight, limiting alcohol consumption, avoiding tobacco, following a healthy diet rich in fruit and vegetables, and exercising regularly can decrease cancer risk [19,20]. Various meta-analyses of prospective cohort studies with relative risk (RR) and corresponding 95% confidence interval (CI) values have demonstrated an inverse association between high consumption of marine omega-3 polyunsaturated fatty acids (PUFAs) [21], dairy products [22], and fiber [23] and the risk of BC. Conversely, a positive association has been found between processed meat consumption [24] and BC risk in prospective observational studies. 

Unhealthy dietary practices are common among young adults [25], whose transition from adolescence to adulthood marks an important phase in establishing behavioral patterns that have a long-lasting impact on overall health and chronic disease risk [26]. Research indicates that this age group face unique challenges in maintaining healthy dietary practices due to various factors such as busy routines, diminished sleep quality, stress from the new autonomy at university, and social pressure concerning body conformity to societal norms [27]. As per the theory of planned behavior [28], dietary practices may be modulated by an individual’s nutrition knowledge and attitude. For example, individuals will have a strong intention to consume the recommended daily amount of fruits and vegetables when they have positive attitudes towards that behavior, feel social pressures from significant others, and perceive that they can eat the recommended amount without difficulty [29]. Addressing gaps in nutrition knowledge, attitudes, and practices (KAP) among young adults early on not only contributes to their immediate health and well-being but also reduces the risk of chronic diseases, including BC, among them and future generations. A few studies have assessed nutrition KAP that may lead to BC risk reduction in the Middle East region, namely in Iran, Jordan, and the kingdom of Saudi Arabia [13,30,31]. The results of these studies highlighted critical gaps and opportunities that could be used to leverage dietary intake to decrease the risk of BC [13,30,31]. Regarding Lebanon, data on knowledge and attitudes towards dietary cancer risk factors are scarce [32]. Some studies assessed knowledge of environmental risk factors for cancer in general [18], or certain types of cancer such as colorectal cancer (CRC) [33], which is screened through methods such as fecal occult blood test, sigmoidoscopy, and colonoscopy [34]. However, to date, no study has examined nutrition KAP that may lead to BC risk reduction in this country. 

It is for this reason that research study was undertaken to assess nutrition KAP that may lead to BC risk reduction among female students at the American University of Beirut (AUB). This study also investigated the possible association between sociodemographic, anthropometric, behavioral, and academic characteristics of female students and their nutrition KAP and examined the determinants of practice. The results may identify evidence-based interventions for university programs geared towards nutrition that may lead to BC risk reduction. 

## 2. Materials and Methods

### 2.1. Study Design and Sampling

A cross-sectional survey was carried out on a convenience sample of AUB female students aged 18 to 25 years who were randomly approached during the period of February to March 2023. Sample size calculations revealed that a minimum of 385 respondents were needed to estimate a conservative prevalence of 50%, with a 95% CI and a margin of error of 5%. The sample size was calculated using the World Health Organization (WHO) sample size calculator [35]. To ensure variation in students’ disciplines and majors, recruitment was conducted on different days of the week, at different campus locations and at different times of the day. Students who had ever been diagnosed with BC or received any treatment for it were excluded from the study. 

### 2.2. Ethical Approval

The AUB Institutional Review Board (IRB) reviewed and approved the protocol of this study under protocol number (SBS-2022-0193). Informed written consent was obtained from all students before data collection. The collected data were processed anonymously without any recognized identifiers, to preserve the students’ privacy and confidentiality. 

### 2.3. Questionnaire

A self-administered questionnaire was used to collect data about nutrition KAP that may lead to BC risk reduction (Appendix A). The questionnaire was constructed following an extensive literature review guided by the Global Cancer Observatory (GLOBOCAN) [36], World Cancer Research Fund (WCRF) and American Institute for Cancer Research (AICR) recommendations [37], as well as the questionnaire validated by Lahiji et al. [13]. Context-specific modifications, including examples of culturally specific food items, were introduced. Prior to the start of the study, a panel of experts in nutrition, epidemiology, public health, and biomedical sciences reviewed and modified the various items included in the questionnaire to ensure its content and face validity. A pilot study was then conducted, after IRB approval, on a convenience sample of 25 AUB female students, in which they were asked to fill out the questionnaires and provide their opinions regarding the clarity and simplicity of the items [38]. Modifications were then introduced accordingly. Data from the pilot study were not included in the analysis. 

The implemented questionnaire consisted of thirty-one KAP questions divided into 18 knowledge, 7 attitude, and 6 practice questions, in addition to general sociodemographic characteristics of the population. The knowledge component included 18 closed-ended questions about non-modifiable and modifiable nutrition-related BC risk factors and the relationship between specific food items and BC. The attitude component included 7 multiple-choice questions (strongly agree, agree, neutral, disagree, and strongly disagree), which obtained information on whether students believe that nutrition and food selection can decrease the risk of BC, that it is necessary to follow a diet to decrease the risk of BC in people under the age of 25, and that being young provides ample time to decrease BC risk. The practice component was a semi-quantitative food frequency questionnaire consisting of 6 items, in which students reported how often they consumed vegetables, fruits, processed meat, red meat, fish, and dairy products. These food items were selected based on the WCRF and AICR, which report food–cancer associations [19,37,39]. To increase the accuracy of portion size estimation, a template depicting serving sizes [40,41,42] and a 2D food portion visual were used [43]. The general characteristics component of the questionnaire collected data on self-reported socio-demographic, anthropometric, behavioral, and academic characteristics, namely students’ age, faculty where they are enrolled, academic major, year at university (sophomore, junior, senior, fourth year, or graduate), marital status, nationality, living situation (living alone, living with parents/relatives, or living with friends or roommates), number of rooms in their house (i.e., their permanent residence) and number of members (including themselves) who are co-residents in their house, family history of BC, height, weight, grade point average (GPA), smoking status, and perceived vulnerability to stress and amount of perceived stress (each on a scale of 1 to 6 [44,45]). The two questions on stress were adapted from the two single-item measures of the ability to handle stress and the amount of stress, which were reported to be reliable and showed validity similar to longer questions [44,45]. Post data collection, an information sheet was provided to students providing recommendations for decreasing BC risk through nutrition.

### 2.4. Scoring of Data

Regarding knowledge, a score of 1 was given to correct answers and 0 for both incorrect and “I don’t know” answers. The minimum possible score was 0 (i.e., the student has no knowledge of the role of nutrition in decreasing the risk of BC), and the maximum possible score was 18 (i.e., the student is very knowledgeable about nutrition KAP that may lead to BC risk reduction). 

Concerning attitudes, a 5-point Likert Scale was used [46]. For the questions indicating a positive attitude toward the role of nutrition in decreasing the risks of BC, the scores ranged between 0, indicating “strongly disagree,” and 4, indicating “strongly agree”. However, for questions indicating a negative attitude toward the role of nutrition in decreasing the risks of BC, scores ranged between 0, indicating “strongly agree,” and 4, indicating “strongly disagree”. The minimum possible score was 0, and the maximum was 28. The validity of this Likert scale was examined using Pearson’s correlation coefficient. 

The scores of the practice component depended on the quantity of food consumed. For fruits and vegetables, a score of 10 was given for consuming ≥400 g/day, a score of 5 for consuming 200–<400 g/day, and a score of 0 for consuming <200 g/day. For red and processed meat, a score of 10 was given to students consuming red meat ≤500 g/week and processed meat <21 g/week, a score of 5 for consuming red meat ≤500 g/week and processed meat ≥21 g but strictly <100 g/week, and a score of 0 for consuming red meat >500 g/week or processed meat ≥100 g/week. For fish, a score of 5, 2.5, or 0 was given to students eating ≥180 g, 90–<180 g, or <90 g of fish per week. For dairy, a score of 5, 2.5, or 0 was given to students consuming ≥2 servings, 1–<2 servings, or <1 serving of dairy per day. A serving of dairy was defined as 1 cup of milk or yogurt or 45 g of natural cheese, according to the U.S. Department of Agriculture MyPlate [47]. The possible scores for practice questions ranged from 0, meaning very poor dietary practices for decreasing the risk of BC, to 30, demonstrating best dietary practices for decreasing the risk of BC. The guidelines used in the scoring scheme for this study were adapted from the WCRF and the AICR Cancer Prevention Recommendations [37]. 

Knowledge and practices were classified as good or poor, and attitudes as positive or negative, using the modified Bloom’s cut-off of 75% of the score [48,49,50,51]. This corresponded to scores of 14, 21, and 23 for KAP, respectively. Knowledge and practices were considered poor and attitudes were deemed negative if they fell below the 75% threshold of their respective scores. 

### 2.5. Statistical Analysis

Data were checked and entered into Statistical Package for the Social Sciences version 27.0 (SPSS Inc., Chicago, IL, USA), with only complete responses being included for data analysis. Accordingly, 356 responses were included in analysis instead of 385. Descriptive statistics were presented to summarize the variables of interest as frequencies and percentages for the categorical variables, and as means and standard deviations (SD) and medians and interquartile ranges (IQR) for the continuous ones. The internal consistency of the data was measured using Cronbach’s α. The Chi-square (χ^2^) test or Fisher’s exact test was used to calculate the association between two categorical variables. Given that none of the continuous variables in the present study adhered to a normal distribution, Mann–Whitney U tests were utilized to compare non-normal continuous variables. The normality of the variables was evaluated through the Kolmogorov–Smirnov and Shapiro–Wilk tests of normality. If both tests are statistically significant, it indicates that the variables of interest do not follow a normal distribution. Simple and multiple logistic regression analyses were used to identify the determinants of KAP. Sociodemographic characteristics served as independent variables, whereas total KAP scores were the dependent variables. Characteristics showing statistical significance in the simple regression were incorporated in the multiple regression models as independent variables. Multiple logistic regression was applied to examine the joint impact of several predictors on KAP levels while adjusting for potential confounding variables included in the model. Results of the logistic regression analyses were reported as odds ratios (OR) with corresponding 95% CI. Moreover, simple linear regression was used, where single components of practice were regressed on the continuous knowledge and attitudes scores. The crowding index, a proxy of socioeconomic status, was calculated as the ratio of the number of people living in the household to the total number of rooms in the house (excluding the kitchen and bathrooms) [52]. Students who live alone or with roommates were grouped together to study whether there were significant differences between their KAP and those of the students who live with parents/relatives. BMI was grouped into three categories: “underweight” (BMI < 18.5 kg/m^2^), “normal weight” (18.5 kg/m^2^ ≤ BMI ≤ 24.9 kg/m^2^), and “overweight and obese” (BMI ≥ 25 kg/m^2^). GPA, which is capped at 4.0 at AUB, was dichotomized into two categories based on a cutoff of 3.2, corresponding to 80/100. According to AUB’s General University Academic Information, “a graduate student is in good standing when her/his graduate grade cumulative average is 80 or above”. The academic major was dichotomized into two categories, health-related vs. non-health-related, to study whether there were significant differences in KAP among their respective students. For all statistical analyses, *p*-values less than 0.05 indicated statistical significance.

## 3. Results

### 3.1. Sample Characteristics

The sociodemographic, anthropometric, behavioral, and academic characteristics of students are presented in Table 1. The mean age was 19.59 ± 1.70 years (18 to 25 years). Most of the students were Lebanese (88.8%), single (97.2%), lived with their parents or relatives (77.8%), never smoked (82.2%), reported their perceived amount of stress as ≥3 (95.5%), and did not have any family history of BC (67.5%). Anthropometric characteristics revealed obesity and overweight in 15.5% of the population studied. Academic characteristics showed that most of the respondents were undergraduates (89.1%) and had a high GPA ≥ 3.2/4.0 (79.4%), and almost half (43.1%) were pursuing a health-related major.

### 3.2. Distribution of KAP among the Sample

Cronbach’s α was >0.7, indicating internal consistency. The percentages of participants’ scores for nutrition KAP that may lead to BC risk reduction are shown in Table 2. Approximately two-thirds of the students had poor knowledge (68.3%) and negative attitudes (65.4%) towards nutrition-related BC risk reduction with the majority (98.0%) having poor practices. The distribution of each of the KAP scores did not follow a normal distribution. The median (IQR) KAP scores in the total sample were 12 (4.0) for knowledge, 20 (4.0) for attitude, and 10 (7.5), for practice, respectively. 

### 3.3. Knowledge

Table 3 assessed the responses to the knowledge questions in the total sample. A high percentage of students provided incorrect answers or indicated a lack of knowledge regarding the protective role of dairy products in decreasing the risk of BC (69.5%) and the association between weight gain and increased risk of BC (60.7%). Moreover, approximately 50% of the students incorrectly answered or did not know that scientific evidence has shown a relationship between diet and BC (49.2%), that being over 50 years old is a risk factor for BC (43.8%), and that the impact of nutrition on BC is intermediate, i.e., around 30% (51.4%). 

On the other hand, most of the students correctly identified that having a family history of BC is a non-modifiable risk factor for this disease (93.5%), that early diagnosis improves survival from BC (94.4%), and that green vegetable intake is protective against BC (79.5%) (Table 3).

### 3.4. Attitudes

Table 4 examined the nutrition attitudes that may lead to BC risk reduction in the sample of AUB female students. The majority of students had positive attitudes towards nutrition being able to decrease the risk of BC (84.6%) and regarding the need to work on decreasing their risk of BC even if there is no family history of the disease (94.9%). Most of the students agreed or strongly agreed that, regardless of the treatment modalities for BC, this disease may cause mortality in these patients (72.2%), and a high number of the students believed that it is necessary to follow a diet to decrease BC risk in people under the age of 25 (56.9%). 

Forty-two percent of students correctly agreed that being young does not provide ample time to decrease BC risk (Table 4). 

### 3.5. Practice 

Table 5 examined nutrition practices that may lead to BC risk reduction in the sample of AUB female students. Most of the students did not meet the recommended amounts of fruits and vegetables (96.9%), fish (87.7%), or dairy (71.4%) and exceeded the permissible amounts of red and processed meat (73.6%). 

Table 6 examined the association between nutrition KAP that may lead to BC risk reduction scores and the sociodemographic, anthropometric, and behavioral characteristics of the students. There was a statistically significant association between academic major and nutrition knowledge (*p* < 0.0001), where a higher percentage of students having high knowledge scores were pursuing health-related majors compared to those having low scores (58.9% vs. 35.8%, respectively). Moreover, a higher GPA was significantly associated with better knowledge scores (*p* = 0.008). There were no significant associations between any other characteristics and knowledge. 

There was a statistically significant association between age, amount of perceived stress, academic year, study major, GPA, and nutrition attitudes that may lead to BC risk reduction. Students having positive attitudes were more likely to be older (*p* = 0.034), have a lower degree of stress (*p* = 0.017), be senior or graduate students (*p* = 0.010), pursuing health-related majors (*p* < 0.0005), and have a higher GPA (*p* = 0.008), compared to those having negative attitudes. All other characteristics had no significant associations with attitudes towards nutrition-related BC risk reduction (Table 6). 

A statistically significant association was noted between major of education and practices, where students having good practice scores were more likely to be pursuing non-health related majors compared to those having low scores. Additionally, having a lower BMI was significantly associated with better practice scores, using the Mann–Whitney U test (*p* = 0.031). All other characteristics had no significant associations with practice (Table 6). 

Table 7 presents the outcomes of both simple and multiple logistic regression analyses for associations between the general characteristics of the students and their levels of nutrition KAP towards BC risk reduction. Simple logistic regression analysis revealed that pursuing a non-health-related major significantly predicted lower levels of knowledge (OR = 0.39, *p* < 0.001), while a higher GPA significantly predicted better knowledge (OR = 2.29, *p* = 0.009). These associations remained significant in the multiple logistic regression model (OR = 0.35, *p ≤* 0.001 and OR = 2.15, *p* = 0.017, respectively).

Positive attitudes were significantly predicted by older age (OR = 1.62, *p* = 0.035), graduate student status (OR = 2.41, *p* = 0.019), and higher GPA (OR = 2.34, *p* = 0.005). Conversely, pursuing a non-health-related major significantly predicted negative attitudes (OR = 0.44, *p* < 0.001). Amount of perceived stress and major of study remained significant predictors of attitudes in the multiple regression model (Table 7).

As for practices, no significant predictors were identified in the simple regression analysis (Table 7). However, BMI approached significance (OR = 0.74, *p* = 0.055). Therefore, no multiple regression analyses were conducted for practices.

Table 8 presents the Spearman correlations between nutrition-related BC risk reduction KAP of AUB female students. There was a statistically significant positive correlation between the overall knowledge and attitude scores (r = 0.39, *p* < 0.0001), where higher knowledge scores were correlated with more positive attitudes towards nutrition-related BC risk reduction. No statistically significant correlation was found between practice and each of the knowledge or attitudes scores.

Simple linear regression of single components of practice on knowledge and attitudes revealed that better knowledge significantly predicted higher intakes of fruits and vegetables (Beta coefficient (95% CI) = 0.047 (0.005–0.089), *p* = 0.029). 

## 4. Discussion

This study is the first to explore KAP related to nutrition and BC risk reduction among Lebanese female youth. Lebanon, a small country in the Middle East, has a distinct context where the median age at BC diagnosis is notably 10 to 15 years earlier than those in the US and Europe, respectively [8,9,10]. The results of this study showed high prevalence of poor knowledge, negative attitudes, and poor practices among the female student population towards nutrition and BC risk reduction. 

Regarding knowledge, the high prevalence of poor knowledge in relation to BC risk reduction revealed in this study could be attributed to the limited presence of national public health awareness campaigns in the country emphasizing the crucial role of nutrition in decreasing the risk of BC. The annual BC screening campaigns initiated by the Ministry of Public health (MOPH) since 2002 have not focused on nutrition [57], thus revealing a gap in addressing the nutrition aspect of BC risk reduction among young women in the country. Additionally, although the Lebanese National Cancer Plan (2023–2028) released by the MOPH mentioned “improving dietary habits and preventing obesity-related cancers” through educational campaigns [58], the extent to which these are implemented remains unknown. The high prevalence of poor nutrition knowledge among educated university students could imply even lower levels of knowledge among the broader “average” female population. Our findings are similar to those reported from other countries such as Malaysia [59] and Nigeria [60], where at least half of surveyed university students had poor knowledge of nutrition-related cancer risk reduction, and consistent with data from Croatia [61] where university students’ knowledge was generally inadequate. The present findings are also consistent with international awareness studies, where data from the US [62] and the United Kingdom (UK) [63] revealed low public awareness regarding several dietary and lifestyle cancer risk factors. 

Answers to specific knowledge questions showed that approximately half of the students did not know that there is an association between diet and BC. This knowledge gap, according to the WCRF, is important, as more than 40% of cancers could be prevented if individuals followed healthy lifestyles including eating a healthy diet [64]. This knowledge gap could also be explained by the fact that, in addition to the lack of nutrition-focused BC awareness campaigns, public and university programs do not highlight this relationship between nutrition and BC and the media provide confusing mixed messages on the role of nutrition in decreasing the risk of BC [62]. Internationally, similar knowledge deficits regarding the diet–cancer relationship were reported from US national studies [65,66] and from the findings of the most recent AICR Cancer Risk Awareness Survey, where more than half of Americans revealed a lack of understanding regarding the role of diet in reducing cancer risk [62]. Regionally, studies from Iran [13] among female university students and from Jordan [30] among adult women confirmed limited knowledge in these population groups on diet and BC risk reduction. 

The present study also demonstrated that a high percentage (69.5%) of students indicated a lack of knowledge regarding the protective role of dairy products in decreasing the risk of BC. This percentage parallels that reported in France, where the majority of the French population (88.2%) did not correctly perceive the protective effect of milk consumption against cancer [67]. Research data reported a statistically significant 5% decrease in risk of premenopausal BC per 200 g of dairy products per day, which could be attributed to the protective effect of dietary calcium [19]. 

Knowledge questions in this study also revealed a knowledge gap regarding the association between weight gain and increased risk of BC. This confirms data from previous studies on nutrition KAP that may lead to BC risk reduction [13,30], where around half of female university students in Iran [13] and Jordanian women [30] did not know that weight gain or overweight and obesity are risk factors for BC. Similarly, data from a national survey in the UK [68], a survey on adults in Ireland [69], and the most recent AICR Cancer Risk Awareness Survey in the US [62] showed that 67%, 68%, and 47% of the participants were not aware of overweight/obesity being cancer risk factors, respectively. In contrast, findings from a population-based study in Denmark and Sweden showed a lower percentage of participants who lacked awareness on the role of obesity in cancer [70]. This better knowledge on relationship between obesity and cancer could be attributed to campaigns addressing lifestyle factors held in the latter countries, which included information about obesity or healthy eating habits and cancer [70]. Scientific evidence underscores the relationship between obesity and BC, showing a 6% increased risk of postmenopausal BC per 5 kg of weight gain [19]. Possible reported mechanisms were related to body fat directly affecting various hormone levels, such as insulin and estrogen, creating an environment that suppresses apoptosis and promotes carcinogenesis, as well as the possibility that the low-grade chronic inflammatory state associated with obesity could be contributing to obesity’s effect on BC [71]. 

Regarding attitudes towards nutrition and BC risk reduction, the majority of students in this study had positive attitudes towards nutrition being able to decrease the risk of BC. This finding is paralleled by data from the U.S., where African-American women strongly agreed that good nutrition can decrease the risk of cancer [72]. Moreover, most of the students agreed they needed to address BC risk reduction even in the absence of a family history of BC, mirroring results from the region [13,30]. 

Assessing practice, this study showed that most of the students did not meet the dietary recommendations for fruits and vegetables, fish, and dairy and exceeded the permissible amounts of red and processed meat. This observed deviation from the recommendations is an issue of concern as evidence has shown that the consumption of fruits, vegetables, fish, and dairy products decreases the risk of BC, while the consumption of processed meat and excess consumption of red meat increase the risk of BC [19,73]. Reported possible mechanisms are that fruits and vegetables are rich in potentially anti-tumorigenic agents, such as carotenoids, vitamins C and E, and other bioactive compounds which may decrease the risk of BC [74,75]. Fruits and vegetables are also rich in fibers which may decrease the risk of BC by improving insulin sensitivity, reducing insulin-like growth factors, and decreasing plasma levels of estrogen [76]. Data from various studies have shown that fish rich in omega-3 PUFAs are associated with decreased BC risk among females [21,77]. Eicosanoids, omega-3 PUFA metabolites, are proposed to act as the modulators of cellular processes through interactions with receptors or by modifying signaling pathways, leading to downregulation of the inflammatory cascade, augmentation of fatty acid (FA) degradation coupled with reduced FA synthesis, and a decrease in the expression of markers, ultimately leading to increased cancer cell death [73]. Dairy products have been found to decrease the risk of BC since they are rich in calcium, vitamin D, and conjugated linoleic acid, which were shown to inhibit cell proliferation and differentiation, suppress angiogenesis, and induce apoptosis and autophagic cell death [78,79,80]. By contrast, this study demonstrated high consumption of red and processed meats in the study population, which is a practice reported in several studies as being associated with the increased risk of BC [56,81]. Possible underlying mechanisms include the “high-fat intake, and/or carcinogens generated through various cooking and processing methods” [82,83].

In this study, a lack of correlation was observed between practice and knowledge scores. This finding is in line with previous studies which highlighted that knowledge is not the only predictor of dietary practice [13,84]. Similarly, data from several countries including the UAE [85] and Brunei [86] have reported the existence of gaps between nutrition-related cancer risk reduction knowledge and practices. Factors influencing nutrition practices could include taste preferences, time and convenience, peer pressure, availability and accessibility to food, higher costs of healthy food items such as fish and dairy products, aggressive marketing of more affordable yet energy-dense foods and beverages, and fast-food culture [84,87]. Thus, the relatively lower costs of fruits and vegetables compared to those of fish and dairy in Lebanon may partly explain why better knowledge only predicted higher intake of fruits and vegetables. Moreover, nearly half of Lebanese households (46%) are food insecure according to the World Food Program [88], and approximately 39% of the college students in Lebanon experience food insecurity, which might explain the poor dietary practices among the participants [89]. 

Investigating associations between knowledge and academic achievements of the sample revealed an association between pursuing health-related majors and better nutrition knowledge scores. This result is paralleled by data from the UK, where university students in the healthcare field of study had higher median scores of nutrition knowledge compared to those in non-healthcare fields of study [90]. The inclusion of nutrition and BC in public health majors was noted as a possible underlying factor for increased awareness. In this study, an association between having higher GPAs and better nutrition knowledge scores was identified, which was also reported to be observed among university students in Jordan [91]. 

Regarding attitudes in this study, age was a contributing factor to positive attitudes, where a positive association between age and positive nutrition attitudes towards BC risk reduction was reported. This could possibly be explained by the increased maturity and added time for knowledge development [92]. Studies from other countries such as Iran [13], assessing nutrition attitudes that may lead to BC risk reduction among female university students, and from Ireland [93], evaluating attitudes towards healthy eating among Irish adults, reported similar positive associations between age and attitudes. The present study also showed that pursuing health-related majors and having a higher GPA were associated with more positive attitudes towards BC risk reduction. This might be explained by the existing positive correlation between nutrition knowledge and attitudes [13]. On the other hand, a negative association between stress and nutrition attitudes was found in this study. Such an association can be perceived in two ways: being more stressed leads to more negative attitudes, and having more negative attitudes towards a topic can lead to more stress.

This study found a significant association between lower BMI and good practice scores, which is consistent with evidence showing that BMI is related to dietary habits [94]. 

### 4.1. Strengths

To our knowledge, this is the first study that tackled nutrition KAP that may lead to BC risk reduction in Lebanon. The questionnaire used had a rigorous development process, was based on an extensive literature review [6] with culture-specific considerations, and was pilot tested. It was also vetted by a panel of experts in nutrition, epidemiology, public health, and biomedical sciences who reviewed and evaluated the various items included in the questionnaire to ensure its validity. Although the survey was self-administered, the practice questionnaire was thoroughly explained to the participants. Moreover, a template depicting serving sizes and a 2D food portion visual were used, to increase the accuracy of portion size estimation. All data were collected by one trained dietitian, ensuring consistency of results.

### 4.2. Limitations

This study had some limitations. The cross-sectional nature of the study prevented the inference of causation. Moreover, the convenience sampling method used, the collection of data from one university, and the high incidence of malnutrition and food insecurity in the country may prevent the generalizability of results to other university female students or to the Lebanese young female population. Socioeconomic status could also influence the results as, in general, AUB students may belong to higher socioeconomic status compared to other young adults in the country. 

## 5. Conclusions

This study highlighted significant gaps in the nutrition-related BC risk reduction KAP among university female students in Lebanon. These findings underscore the need to develop and implement appropriate, well-designed, and focused BC and nutrition awareness campaigns to enhance nutrition-related BC knowledge and attitudes, and to promote dietary practices that may decrease BC risk among this young female population. BC-targeted nutrition education programs that provide evidence-based information could address misconceptions, change negative attitudes to positive ones, further enhance positive attitudes, and translate them into sustained good nutrition practices. Future studies that consider a broader age range and larger scale are needed to unravel the predictors of nutrition-related BC risk reduction practices in the Lebanese population. 

## Figures and Tables

**Table 1:** General characteristics of the students (*n* = 356).

Students’ Characteristics	*n* (%)
Age (years) ^a,^*	
≤19	208 (59.3)
>19	143 (40.7)
Marital status *	
Married/Engaged	10 (2.8)
Single	345 (97.2)
**Living situation**	
Live alone or with roommates	79 (22.2)
Live with parents/relatives	277 (77.8)
**Nationality**	
Lebanese	316 (88.8)
Non-Lebanese	40 (11.2)
Crowding index *	
<1	146 (42.2)
≥1	200 (57.8)
Smoking status *	
Ever Smoker	62 (17.8)
Never Smoker	290 (82.2)
Perceived Vulnerability to Stress *	
<3	79 (22.4)
≥3	274 (77.6)
Amount of perceived stress *	
<3	16 (4.5)
≥3	337 (95.5)
Family history of BC *	
No	239 (67.5)
Yes	115 (32.5)
BMI classification ^b,^*	
Underweight	37 (11.2)
Normal	242 (73.3)
Overweight and Obese	51 (15.5)
Academic year *	
Sophomore	130 (37.1)
Junior	112 (32.0)
Senior	54 (15.4)
Fourth year	16 (4.6)
Graduate	38 (10.9)
Major *	
Health-related	153 (43.1)
Non-health-related	202 (56.9)
GPA (capped at 4.0) ^c,^*	
<3.2 (corresponding to 80/100)	61(20.6)
≥3.2 (corresponding to 80/100)	235 (79.4)

^a^ Mean age ± SD: 19.59 ± 1.70 years, Median age (IQR): 19.00 (3.00) years; ^b^ Mean BMI ± SD: 22.32 ± 3.28 kg/m^2^, Median BMI (IQR): 22.06 (4.07) kg/m^2^; ^c^ Mean GPA ± SD: 3.49 ± 0.44, Median GPA (IQR): 3.60 (0.64); GPA at AUB is capped at 4.0, as per AUB’s general university academic information. * Total numbers in this table are not always equal to 356 (number of participants in this study) because of missing answers.

**Table 2:** Percent of population with poor scores for knowledge and practice and negative scores for attitude among female AUB students aged 18 to 25.

Variables	Evaluations and Judgments	*n* (%)
Knowledge ^a^	Poor	243 (68.3)
	Good	113 (31.7)
Attitude ^b^	Negative	233 (65.4)
	Positive	123 (34.6)
Practices ^c^	Poor	349 (98.0)
	Good	7 (2.0)

^a^ Poor knowledge was defined as scores <75%, corresponding to 14. ^b^ Negative attitudes were defined as scores <75%, corresponding to 21. ^c^ Poor practices were defined as scores <75%, corresponding to 23.

**Table 3:** Evaluation of knowledge questionnaire (*n* = 356).

Knowledge Questions ^a^	Correct Answer	Answered Correctly *n* (%)	Answered Incorrectly or I Don’t Know. *n* (%)
Q1. Has scientific evidence shown a relationship between diet and breast cancer? * [19]	Yes	180 (50.8)	174 (49.2)
Q2. Is being a female a risk factor for breast cancer? [14]	Yes	255 (71.6)	101 (28.4)
Q3. Is being over 50 years old a risk factor for breast cancer? [14]	Yes	200 (56.2)	156 (43.8)
Q4. Is having a family history of breast cancer a risk factor for breast cancer? [14]	Yes	333 (93.5)	23 (6.5)
Q5. What is the impact of nutrition on breast cancer? * [53,54]	Intermediate (around 30%)	172 (48.6)	182 (51.4)
Q6. Does early diagnosis of breast cancer improve survival? * [19]	Yes	334 (94.4)	20 (5.6)
Q7. Do contraceptive pills increase the risk of developing breast cancer? [19]	Yes	164 (46.1)	192 (53.9)
Q8. Does smoking increase the risk of developing breast cancer? * [14]	Yes	265 (74.6)	90 (25.4)
Q9. Does weight gain increase the risk of developing breast cancer? * [19]	Yes	139 (39.3)	215 (60.7)
Q10. Does being physically active increase the risk of developing breast cancer? [19]	No	270 (75.8)	86 (24.2)
Q11. Green vegetable intake in relation to breast cancer risk [19]	Protective	283 (79.5)	73 (20.5)
Q12. Fruit intake in relation to breast cancer risk * [55]	Protective	267 (76.3)	83 (23.7)
Q13. Consumption of processed red meat (e.g., sausages, Mortadella, Makanik, hot dogs, etc.) in relation to breast cancer risk * [56]	Harmful	209 (60.2)	138 (39.8)
Q14. High consumption of red meat in relation to breast cancer risk * [56]	Harmful	217 (61.6)	135 (38.4)
Q15. Consumption of fish (omega 3) in relation to breast cancer risk * [21]	Protective	243 (69.4)	107 (30.6)
Q16. Consumption of dairy products in relation to breast cancer risk * [19]	Protective	105 (30.5)	239 (69.5)
Q17. Alcohol consumption in relation to breast cancer risk * [19]	Harmful	281 (79.8)	71 (20.2)
Q18. Foods containing carotenoids (e.g., bell peppers, broccoli, cantaloupe, carrots, kale, mangoes, oranges, spinach, tomatoes, etc.) in relation to breast cancer risk [19]	Protective	240 (67.4)	116 (32.6)

* Total numbers in this table are not always equal to 356 (number of participants in this study) because of missing answers. ^a^ Numbers in square brackets next to each question are the references for the correct answers.

**Table 4:** Evaluation of nutrition attitudes that may lead to BC risk reduction from questionnaire administered to sample of AUB female students (*n* = 356).

Attitude	Strongly Agree *n* (%)	Agree *n* (%)	Neutral *n* (%)	Disagree *n* (%)	Strongly Disagree *n* (%)
I believe that nutrition can decrease the risk of BC.	89 (25.0)	212 (59.6)	49 (13.8)	4 (1.1)	2 (0.6)
I need to work on decreasing the risk of BC even if I don’t have a family history of BC.	207 (58.1)	131 (36.8)	17 (4.8)	1 (0.3)	0 (0.0)
I believe that it is necessary to follow a diet to decrease BC risk in people under the age of 25 *.	65 (18.3)	137 (38.6)	118 (33.2)	34 (9.6)	1 (0.3)
I believe that, despite the treatment modalities available for patients with BC, this disease can cause the death of these patients.	95 (26.7)	162 (45.5)	68 (19.1)	25 (7.0)	6 (1.7)
I believe that adherence to a healthy diet by a person reduces the risk of developing BC in the next generation.	60 (16.9)	163 (45.8)	109 (30.6)	22 (6.2)	2 (0.6)
I believe that food selection does not decrease the risk of BC *.	6 (1.7)	19 (5.4)	93 (26.2)	196 (55.2)	41 (11.5)
I believe that I am young, so I have a lot of time to decrease my risk of BC.	42 (11.8)	83 (23.3)	81 (22.8)	104 (29.2)	46 (12.9)

* Total numbers in this table are not always equal to 356 (number of participants in this study) because of missing answers.

**Table 5:** Nutrition practices that may lead to BC risk reduction in the sample of AUB female students (*n* = 356).

Nutrition Practices	Average Consumption Mean ± SD	Meeting Recommendation	Not Meeting Recommendation
**Consumption of fruits and vegetables** (Recommendation: ≥400 g/day)	152 ± 104 g/day	11 (3.1)	345 (96.9)
**Consumption of red and processed meat** * (Recommendation: Red meat ≤500 g/week and processed meat <21 g/week)	Red meat: 312.2 ± 350.6 g/week Processed meat: 146.7 ± 311.1 g/week	93 (26.4)	259 (73.6)
**Consumption of fish** * (Recommendation: ≥180 g/week)	81 ± 198 g/week	43 (12.3)	308 (87.7)
**Consumption of dairy** *^,**a**^ (Recommendation: ≥2 servings/day)	0.1 ± 0.3 servings/day	100 (28.6)	250 (71.4)

* Total numbers in this table are not always equal to 356 (number of participants in this study) because of missing answers. ^a^ A serving of dairy is defined as 1 cup of milk or yogurt or 45 g of natural cheese [47].

**Table 6:** Association between nutrition-related BC risk reduction knowledge, attitudes, and practice scores and general characteristics of AUB female students (*n* = 356).

Characteristics	Knowledge ^i^		*p*-Value *	Attitudes ^ii^		*p*-Value *	Practices ^iii^		*p*-Value *
	Poor	Good		Negative	Positive		Poor	Good	
**Age (years)**, *n* (%)			0.069 ^a^			**0.034 ^a^**			0.449 ^b^
≤ 19	150 (62.5)	58 (52.3)		145 (63.3)	63 (51.6)		205 (59.6)	3 (42.9)	
>19	90 (37.5)	53 (47.7)		84 (36.7)	59 (48.4)		139 (40.4)	4 (57.1)	
** Mean age ± SD **	19.49 ± 1.61	19.81 ± 1.86	0.175 ^d^	19.45 ± 1.64	19.85 ± 1.78	**0.027 ^d^**	19.58 ± 1.70	20.00 ± 1.73	0.448 ^d^
** Median age (IQR) **	19.00 (2.00)	19.00 (3.00)		19.00 (2.00)	19.00 (3.00)		19.00 (2.00)	20.00 (4.00)	
**Marital status**, *n* (%)			0.300 ^b^			>0.99 ^b^			>0.99 ^b^
Married/Engaged	5 (2.1)	5 (4.4)		7 (3.0)	3 (2.4)		10 (2.9)	0 (0.0)	
Single	237 (97.9)	108 (95.6)		225 (97.0)	120 (97.6)		338 (97.1)	7 (100.0)	
**Living situation**, *n* (%)			0.423 ^a^			0.126 ^a^			0.654 ^b^
Live alone or with roommates	51 (21.0)	28 (24.8)		46 (19.7)	33 (26.8)		77 (22.1)	2 (28.6)	
Live with parents/relatives	192 (79.0)	85 (75.2)		187 (80.3)	90 (73.2)		272 (77.9)	5 (71.4)	
**Nationality**, *n* (%)			0.913 ^a^			0.913 ^a^			>0.99 ^b^
Lebanese	216 (88.9)	100 (88.5)		216 (88.9)	100 (88.5)		309 (88.5)	7 (100.0)	
Non-Lebanese	27 (11.1)	13 (11.5)		27 (11.1)	13 (11.5)		40 (11.5)	0 (0.0)	
**Crowding index**, *n* (%)			0.863 ^a^			0.863 ^a^			0.461 ^b^
<1	98 (41.9)	48 (42.9)		98 (41.9)	48 (42.9)		142 (41.9)	4 (57.1)	
≥1	136 (58.1)	64 (57.1)		136 (58.1)	64 (57.1)		197 (58.1)	3 (42.9)	
**Smoking status**, *n* (%)			0.234 ^a^			0.234 ^a^			0.36 ^b^
Ever Smoker	47 (19.5)	16 (14.3)		47 (19.5)	16 (14.3)		63 (18.2)	0 (0.0)	
Never Smoker	194 (80.5)	96 (85.7)		194 (80.5)	96 (85.7)		283 (81.8)	7 (100.0)	
**Perceived Vulnerability to Stress**, *n* (%)			0.750 ^a^			0.682 ^a^			>0.99 ^b^
<3	53 (21.9)	26 (23.4)		53 (23.0)	26 (21.1)		78 (22.5)	1 (14.3)	
≥3	189 (78.1)	85 (76.6)		177 (77.0)	97 (78.9)		268 (77.5)	6 (85.7)	
**Amount of perceived stress**, *n* (%)			0.986 ^a^			**0.017 ^a^**			>0.99 ^b^
<3	11 (4.5)	5 (4.5)		6 (2.6)	10 (8.1)		16 (4.6)	0 (0.0)	
≥3	231 (95.5)	106 (95.5)		224 (97.4)	113 (91.9)		330 (95.4)	7 (100.0)	
**Family history of BC**, *n* (%)			0.881 ^a^			0.881 ^a^			0.686 ^b^
No	164 (67.8)	75 (67.0)		164 (67.8)	75 (67.0)		235 (67.7)	4 (57.1)	
Yes	78 (32.2)	37 (33.0)		78 (32.2)	37 (33.0)		112 (32.3)	3 (42.9)	
**BMI classification**, *n* (%)			0.924 ^c^			0.924 ^c^			>0.99 ^c^
Underweight	24 (10.8)	13 (12.0)		24 (10.8)	13 (12.0)		36 (11.1)	1 (14.3)	
Normal	165 (74.3)	77 (71.3)		165 (74.3)	77 (71.3)		237 (73.4)	5 (71.4)	
Overweight and Obese	33 (14.9)	18 (16.7)		33 (14.9)	18 (16.7)		50 (15.5)	1 (14.3)	
**Mean BMI ± SD**	22.25 ± 3.21	22.46 ± 3.44	0.701 ^d^	22.41 ± 3.25	22.16 ± 3.34	0.268 ^d^	22.37 ± 3.28	20.00 ± 2.76	**0.031 ^d^**
**Median BMI (IQR)**	22.04 (4.10)	22.12 (4.01)		22.19 (4.17)	21.79 (3.51)		22.10 (4.02)	19.26 (1.40)	
**Academic year**, *n* (%)			0.200 ^c^			**0.010 ^c^**			0.659 ^c^
Sophomore	89 (37.6)	41 (36.3)		89 (39.2)	41 (33.3)		128 (37.3)	2 (28.6)	
Junior	80 (33.8)	32 (28.3)		79 (34.8)	33 (26.8)		110 (32.1)	2 (28.6)	
Senior	35 (14.8)	19 (16.8)		31 (13.7)	23 (18.7)		52 (15.2)	2 (28.6)	
Fourth year	12 (5.1)	4 (3.5)		10 (4.4)	6 (4.9)		16 (4.7)	0 (0.0)	
Graduate	21 (8.9)	17 (15.0)		18 (7.9)	20 (16.3)		37 (10.8)	1 (14.3)	
**Major**, *n* (%)			**<0.0001 ^a^**			**<0.0005 ^a^**			**0.021 ^b^**
Health-related	87 (35.8)	66 (58.9)		84 (36.2)	69 (56.1)		153 (44.0)	0 (0.0)	
Non-health-related	156 (64.2)	46 (41.1)		148 (63.8)	54 (43.9)		195 (56.0)	7 (100.0)	
**GPA (capped at 4.0)**, *n* (%)			0.076 ^a^			**0.005 ^a^**			0.351 ^b^
<3.2	47 (23.5)	14 (14.6)		48 (25.7)	13 (11.9)		61 (21.1)	0 (0.0)	
≥3.2	153 (76.5)	82 (85.4)		139 (74.3)	96 (88.1)		228 (78.9)	7 (100.0)	
**Mean GPA ± SD**	3.45 ± 0.46	3.59 ± 0.39	**0.008 ^d^**	3.44 ± 0.47	3.59 ± 0.37	**0.008 ^d^**	3.49 ± 0.44	3.77 ± 0.20	0.099 ^d^
**Median GPA (IQR)**	3.50 (0.60)	3.70 (0.64)		3.50 (0.65)	3.70 (0.58)		3.60 (0.63)	3.70 (0.40)	

^i^ Knowledge score is based on the number of correct knowledge answers (scores can range between 0 and 18; the higher the score, the higher the knowledge). Good knowledge score was defined as ≥75%, which corresponds to 14. ^ii^ Attitude score is based on the number of positive attitude statements (scores can range between 0 and 28; the higher the score, the more positive the attitude towards the role of nutrition in decreasing BC risk). Positive attitude score was defined as ≥75%, which corresponds to 21. ^iii^ Practice score is based on the quantity of food consumed (scores can range between 0 and 30; the higher the score, the better the practice). Good practices were defined as ≥75%, which corresponds to 23. * Bolded numbers indicate statistical significance (*p*-value < 0.05). ^a^ Chi-square test; ^b^ Fisher’s exact test; ^c^ Linear-by-Linear association; ^d^ Mann–Whitney U Test.

**Table 7:** Simple and multiple logistic regression for associations of general characteristics of AUB female students (*n* = 356) with levels of nutrition knowledge, attitudes, and practices towards BC risk reduction.

Characteristics	Knowledge **				Attitudes ***				Practice ****	
	Simple Logistic Regression		Multiple Logistic Regression		Simple Logistic Regression		Multiple Logistic Regression		Simple Logistic Regression	
	OR (95%CI)	*p*-Value *	OR (95%CI)	*p*-Value *	OR (95%CI)	*p*-Value *	OR (95%CI)	*p*-Value *	OR (95%CI)	*p*-Value *
**Age (years)**		0.070				**0.035**		0.541		0.381
≤19	1				1		1		1	
>19	1.52 (0.97–2.4)				1.62 (1.04–2.52)		1.31 (0.55–3.16)		1.97 (0.43–8.92)	
**Marital status**		0.222				0.754			(–)	
Married/Engaged	1				1					
Single	0.46 (0.13–1.61)				1.24 (0.32–4.9)					
**Living situation**		0.423				0.127				0.683
Live alone or with roommates	1				1				1	
Live with parents/relatives	0.81 (0.48–1.37)				0.67 (0.4–1.12)				0.71 (0.13–3.72)	
**Nationality**		0.913				0.772			(–)	
Lebanese	1				1					
Non-Lebanese	1.04 (0.51–2.1)				0.9 (0.45–1.82)					
Crowding index		0.863				0.423				0.423
<1	1				1				1	
≥1	0.96 (0.61–1.51)				0.83 (0.53–1.3)				0.83 (0.53–1.3)	
**Smoking status**		0.235				0.218			(–)	
Ever Smoker	1				1					
Never Smoker	1.45 (0.78–2.7)				0.7 (0.4–1.23)					
**Perceived Vulnerability to Stress**		0.750				0.682				0.608
<3	1				1				1	
≥3	0.92 (0.54–1.56)				1.12 (0.66–1.9)				1.75 (0.21–14.72)	
**Amount of perceived stress**		0.986				**0.024**		**0.018**	(–)	
<3	1				1		1			
≥3	1.01 (0.34–2.98)				0.3 (0.11–0.85)		0.18 (0.04–0.74)			
**Family history of BC**		0.881				0.641				0.557
No	1				1				1	
Yes	1.04 (0.64–1.67)				0.89 (0.56–1.43)				1.57 (0.35–7.15)	
**BMI**	1.02 (0.95–1.09)	0.592			0.98 (0.91–1.05)	0.512			0.74 (0.55–1.01)	0.055
**Academic year**		0.415				**0.074**		0.743		0.919
Sophomore	1				1		1			
Junior	0.87 (0.5–1.51)	0.616			0.91 (0.52–1.57)	0.727	0.74 (0.38–1.44)	0.381	1.16 (0.16–8.4)	0.881
Senior	1.18 (0.6–2.3)	0.631			1.61 (0.84–3.01)	0.153	1.31 (0.44–3.94)	0.627	2.46 (0.34–17.94)	0.374
Fourth year	0.72 (0.22–2.38)	0.594			1.3 (0.44–3.83)	0.631	1.02 (0.26–4.51)	0.916	(–)	
Graduate	1.76 (0.84–3.68)	0.135			2.41 (1.15–5.04)	**0.019**	1.30 (0.39–4.37)	0.666	1.73 (0.15–19.61)	0.658
**Major**		**<0.001**		**<0.001**		**<0.001**		**<0.001**	(–)	
Health-related	1		1		1		1			
Non-health-related	0.39 (0.25–0.61)		0.35 (0.21–0.58)		0.44 (0.28–0.69)		0.37 (0.22–0.63)			
**GPA**	2.29 (1.23–4.25)	**0.009**	2.15 (1.15–4.03)	**0.017**	2.34 (1.29–4.26)	**0.005**	1.84 (0.96–3.53)	0.067	9.72 (0.62–151.9)	0.105

* Bolded numbers indicate statistical significance (*p*-value < 0.05). ** Knowledge score is based on the number of correct knowledge answers (scores can range between 0 and 18; the higher the score, the higher the knowledge). Good knowledge score was defined as ≥75%, which corresponds to 14. *** Attitude score is based on the number of positive attitude statements (scores can range between 0 and 28; the higher the score, the more positive the attitude towards the role of nutrition in decreasing BC risk). Positive attitude score was defined as ≥75%, which corresponds to 21. **** Practice score is based on the quantity of food consumed (scores can range between 0 and 30; the higher the score, the better the practice). Good practices were defined as ≥75%, which corresponds to 23. (–) *n* = 0 in one of the groups

**Table 8:** Spearman correlations between nutrition knowledge, attitudes, and practice that may lead to BC risk reduction among AUB female students.

	Overall Knowledge		Overall Attitude		Overall Practice	
	r-Coefficient	*p*-Value	r-Coefficient	*p*-Value	r-Coefficient	*p*-Value *
Overall knowledge	-					
Overall attitude	0.387	**<0.0001**	-			
Overall practice	0.076	0.152	0.028	0.601	-	

* Bolded numbers indicate statistical significance (*p*-value < 0.05).

## Data Availability

The data presented in this study are available on request from the corresponding author. The data are not publicly available due to privacy reasons.

## Appendix A

Questionnaire: Assessing Nutrition Knowledge, Attitudes, and Practice for Breast Cancer Prevention among Female Students at the American University of Beirut.

### Appendix A.1. Assessing Knowledge

1- Has scientific evidence shown a relationship between diet and breast cancer?
  ☐ Yes
  ☐ No
  ☐ I do not know
2- What is/are the risk factors for breast cancer? (You can tick more than one)
  ☐ Being a female
  ☐ Being over 50 years old
  ☐ Having a family history of breast cancer
  ☐ None of the above
  ☐ I do not know
3- What is the impact of nutrition on breast cancer?
  ☐ Low (up to 5%)
  ☐ Intermediate (around 30%)
  ☐ High (around 100%)
  ☐ It does not have an impact
  ☐ I do not know
4- Does early diagnosis of breast cancer improve survival?
  ☐ Yes
  ☐ No
  ☐ I do not know
5- Do these factors increase the risk of developing breast cancer?
  	Yes	No	I Do Not Know
  Contraceptive pills			
  Smoking			
  Weight Gain			
  Being physically active			
6- Foods in relation to breast cancer prevention
  	Protective	Harmful	Not Related	I Do Not Know
  Green Vegetable intake				
  Fruit intake				
  Consumption of processed red meat (e.g., sausages, Mortadella, Makanik, hot dog, etc.)				
  High consumption of red meat				
  Consumption of fish (omega 3)				
  Consumption of dairy products				
  Alcohol consumption				
  Foods containing carotenoids (e.g., bell peppers, broccoli, cantaloupe, carrots, kale, mangoes, oranges, spinach, tomatoes, etc.)				

### Appendix A.2. Attitude Questionnaire

	Strongly Agree	Agree	Neutral	Disagree	Strongly Disagree
1- I believe that nutrition can decrease the risk of breast cancer.					
2- I need to work on breast cancer prevention even if I don’t have a family history of breast cancer.					
3- I believe that it is necessary to follow a diet to prevent breast cancer in people under the age of 25.					
4- I believe that food selection does not decrease the risk of breast cancer.					
5- I believe that, despite the treatment modalities available for patients with breast cancer, this disease can cause the death of these patients.					
6- I believe that adherence to a healthy diet by a person reduces the risk of developing breast cancer in the next generation.					
7- I believe that I am young, so I have a lot of time to prevent breast cancer.					

### Appendix A.3. Practice Questionnaire

Please indicate your usual intake of each of the following food items per Day, Week, or Month.

**Food Item**	**Serving Size**	**Day**	**Week**	**Month**	**Rarely/Never**
Vegetables (raw and cooked)	1 cup of raw or cooked vegetables or 2 cups of raw green leafy vegetables				
Fruits	1 fruit or 1 cup of fruits				
Processed Meat (Sausages, Makanik, Basterma, and Mortadella)	90 g				
Red meat (mutton, beef, minced)	90 g				
Fish	90 g				
Low fat dairy products (milk, yogurt, natural cheese)	1 cup milk or yogurt or 45 g of natural cheese				

### Appendix A.4. Anthropometric, Behavioral, Sociodemographic, and General Characteristics Questionnaire

1- Age (years) _____________
2- Faculty of Education
  ○ FAFS
  ○ FAS
  ○ OSB
  ○ MSFEA
  ○ FHS
  ○ HSON
  ○ FM
3- Major _____________
4- Academic year
  ○ Sophomore
  ○ Junior
  ○ Senior
  ○ Fourth year
  ○ Master’s
5- Marital status
  ○ Married
  ○ Engaged
  ○ Single
6- Nationality: --------------
7- Which statement best describes your living situation?
  - Live alone
  - Live with parents/relatives
  - Live with friends/roommates
8- How many rooms are in your house, i.e., your permanent residence? (Excluding the kitchen and bathrooms) _____________
9- How many members (including yourself) are co-residents in your house?_____________
10- Do you have any family history of breast cancer?
  ○ No
  ○ Yes
  If Yes: please specify
  ○ First degree relatives (parents and siblings)
  ○ Second degree relatives (uncles–aunts–grandparents)
11- Height (cm) _____________
12- Weight (kg) _____________
13- GPA (capped at 4.0) _____________
14- Do you smoke (any kinds of smoking, cigarettes, shisha, e-cigarettes, IQOS, or vape)?
  ○ Everyday
  ○ 3 times per week
  ○ 3 times per month
  ○ Never
15- On a scale of 1 to 6, how would you rate your ability to handle stress? (from 1 for “I can shake off stress” to 6 for “stress eats away at me”) _____________
16- In the past year, how would you rate the amount of stress in your life (at your residence and at university)? (from 1 for “no stress” to 6 for “extreme stress”) _____________
  If you perceived any emotional disturbances while filling the questionnaire, don’t hesitate to contact AUB counseling center:
  Tel.: +961 1 350000
  Ext.: 3196 (only during working hours)
  Email: counselingcenter@aub.edu.lb

## References

[1] Arnold M., Morgan E., Rumgay H., Mafra A., Singh D., Laversanne M., Vignat J., Gralow J.R., Cardoso F., Siesling S. (2022). Current and Future Burden of Breast Cancer: Global Statistics for 2020 and 2040. Breast.

[2] Union for International Cancer Control (2020). *GLOBOCAN* 2020: New Global Cancer Data. https://www.uicc.org/news/globocan-2020-new-global-cancer-data.

[3] Wilkinson L., Gathani T. (2022). Understanding Breast Cancer as a Global Health Concern. Br. J. Radiol..

[4] Global Cancer Observatory (2024). Cancer Mortality in Females in 2022. https://gco.iarc.fr/today/en/dataviz/pie?mode=cancer&group_populations=1&types=1&sexes=2.

[5] Global Cancer Observatory (2020). Breast Fact. Sheet. https://gco.iarc.fr/today/data/factsheets/cancers/20-Breast-fact-sheet.pdf.

[6] World Health Organization (2023). Breast Cancer. https://www.who.int/news-room/fact-sheets/detail/breast-cancer.

[7] Global Cancer Observatory (2024). Lebanon Fact Sheet. https://gco.iarc.who.int/media/globocan/factsheets/populations/422-lebanon-fact-sheet.pdf.

[8] Elias F., Rabah H., Salih M., Boushnak M., Said C. (2021). Effectiveness of Breast Cancer Screening Campaigns from 2012 to 2017 by Analysis of Stage at Diagnosis, Lebanon. East. Mediterr. Health J..

[9] National Cancer Institute Cancer Stat Facts: Female Breast Cancer. https://seer.cancer.gov/statfacts/html/breast.html.

[10] Anwar S.L., Raharjo C.A., Herviastuti R., Dwianingsih E.K., Setyoheriyanto D., Avanti W.S., Choridah L., Harahap W.A., Darwito, Aryandono T. (2019). Pathological Profiles and Clinical Management Challenges of Breast Cancer Emerging in Young Women in Indonesia: A Hospital-based Study. BMC Women’s Health.

[11] Sibai A.M., Hwalla N., Adra N., Rahal B. (2003). Prevalence and Covariates of Obesity in Lebanon: Findings from the First Epidemiological Study. Obes. Res..

[12] Fares M.Y., Salhab H.A., Khachfe H.H., Khachfe H.M. (2019). Breast cancer epidemiology among Lebanese women: An 11-year analysis. Medicina.

[13] Raji Lahiji M., Dehdari T., Shokouhi Shoormasti R., Hosseini A.F., Navaei M., Zarrati M. (2019). Nutrition Knowledge, Attitudes, and Practice towards Breast Cancer Prevention among the Female Population of Iran University of Medical Science Students. Nutr. Cancer.

[14] Centers for Disease Control and Prevention (2022). What Are the Risk Factors for Breast Cancer?. https://www.cdc.gov/cancer/breast/basic_info/risk_factors.htm.

[15] Anand P., Kunnumakkara A.B., Sundaram C., Harikumar K.B., Tharakan S.T., Lai O.S., Sung B., Aggarwal B.B. (2008). Cancer is a Preventable Disease that Requires Major Lifestyle Changes. Pharm. Res..

[16] (2023). Breast Cancer Facts and Statistics. https://www.breastcancer.org/facts-statistics.

[17] Möller S., Mucci L.A., Harris J.R., Scheike T., Holst K., Halekoh U., Adami H.O., Czene K., Christensen K., Holm N.V. (2016). The Heritability of Breast Cancer among Women in the Nordic Twin Study of Cancer. Cancer Epidemiol. Biomark. Prev..

[18] Kabalan M., El-Hajj M., Khachman D., Awada S., Rachidi S., Al-Hajje A., Ajrouche R. (2021). Public Awareness of Environmental Risk Factors of Cancer and Attitude towards its Prevention among the Lebanese General Population. J. Prev. Med. Hyg..

[19] World Cancer Research Fund, American Institue for Cancer Research (2018). Breast Cancer. https://www.wcrf.org/wp-content/uploads/2021/02/Breast-cancer-report.pdf.

[20] Brennan S.F., Cantwell M.M., Cardwell C.R., Velentzis L.S., Woodside J.V. (2010). Dietary Patterns and Breast Cancer Risk: A Systematic Review and Meta-Analysis. Am. J. Clin. Nutr..

[21] Zheng J.-S., Hu X.-J., Zhao Y.-M., Yang J., Li D. (2013). Intake of Fish and Marine N-3 Polyunsaturated Fatty Acids and Risk of Breast Cancer: Meta-analysis of Data from 21 Independent Prospective Cohort Studies. BMJ.

[22] Dong J.-Y., Zhang L., He K., Qin L.-Q. (2011). Dairy consumption and risk of breast cancer: A meta-analysis of prospective cohort studies. Breast Cancer Res. Treat..

[23] Chen S., Chen Y., Ma S., Zheng R., Zhao P., Zhang L., Liu Y., Yu Q., Deng Q., Zhang K. (2016). Dietary Fibre Intake and Risk of Breast Cancer: A Systematic Review and Meta-Analysis of Epidemiological Studies. Oncotarget.

[24] Farvid M.S., Stern M.C., Norat T., Sasazuki S., Vineis P., Weijenberg M.P., Wolk A., Wu K., Stewart B.W., Cho E. (2018). Consumption of Red and Processed Meat and Breast Cancer Incidence: A Systematic Review and Meta-Analysis of Prospective Studies. Int. J. Cancer.

[25] Allman-Farinelli M., Partridge S.R., Roy R. (2016). Weight-Related Dietary Behaviors in Young Adults. Curr. Obes. Rep..

[26] Small M., Bailey-Davis L., Morgan N., Maggs J. (2013). Changes in Eating and Physical Activity Behaviors Across Seven Semesters of College: Living On or Off Campus Matters. Health Educ. Behav..

[27] Alves R., Petitjean H., Druzhinenko-Silhan D. (2024). Psychological approaches to obesity in young adults: State of the art. Front. Nutr..

[28] McDermott M.S., Oliver M., Simnadis T., Beck E.J., Coltman T., Iverson D., Caputi P., Sharma R. (2015). The Theory of Planned Behaviour and Dietary Patterns: A Systematic Review and Meta-Analysis. Prev. Med..

[29] McDermott M.S., Oliver M., Svenson A., Simnadis T., Beck E.J., Coltman T., Iverson D., Caputi P., Sharma R. (2015). The Theory of Planned Behaviour and Discrete Food Choices: A Systematic Review and Meta-Analysis. Int. J. Behav. Nutr. Phys. Act..

[30] Ajlouni B., Al-Nabulsi A., Issa S.N., Farha R.A., Wafa’a Ta’an R., Saleh S.A., Itani R., Tawalbeh R., Mukattash T. (2023). Evaluating Nutrition-related Knowledge, Attitudes, and Practices for the Prevention of Breast Cancer among Women in Jordan. Jordan J. Nurs. Res..

[31] Shamsuddeen S.B., Ansari A., Ali M.S. (2023). Nutrition Knowledge, Attitude, Practice towards Breast Cancer Prevention among the Female Students of University of Hail, Saudi Arabia. Med. Sci. Discov..

[32] Hatem G., Ghanem D., Kellen E., AlZaim I., Goossens M. (2021). Knowledge and Beliefs of Cancer Risk Factors and Early Cancer Symptoms in Lebanon: A Cross-Sectional Survey among Adults in the Community. Cancer Control.

[33] Tfaily M.A., Naamani D., Kassir A., Sleiman S., Ouattara M., Moacdieh M.P., Jaffa M.A. (2019). Awareness of Colorectal Cancer and Attitudes towards its Screening Guidelines in Lebanon. Ann. Glob. Health.

[34] National Cancer Institute (2023). Colorectal Cancer Screening (PDQ®)–Health Professional Version. https://www.cancer.gov/types/colorectal/hp/colorectal-screening-pdq.

[35] Sample Size Calculator. https://cdn.who.int/media/docs/default-source/ncds/ncd-surveillance/steps/sample-size-calculator.xls.

[36] Global Cancer Observatory Cancer Causes. https://gco.iarc.fr.

[37] Standardized Scoring System to Assess Adherence to WCRF/AICR Cancer Prevention Recommendations. https://epi.grants.cancer.gov/wcrf-aicr-score/.

[38] Tsang S., Royse C.F., Terkawi A.S. (2017). Guidelines for Developing, Translating, and Validating a Questionnaire in Perioperative and Pain Medicine. Saudi J. Anaesth..

[39] American Institute for Cancer Research (2015). Fish and Cancer Risk: 4 Things You Need to Know. https://www.aicr.org/news/fish-and-cancer-risk-4-things-you-need-to-know/.

[40] Academy of Nutrition and Dietetics (2020). Serving Size vs. Portion Size: Is there a Difference?. https://www.eatright.org/health/wellness/nutrition-panels-and-food-labels/serving-size-vs-portion-size-is-there-a-difference.

[41] WebMD (2019). Portion Size Guide. https://img.webmd.com/dtmcms/live/webmd/consumer_assets/site_images/media/pdf/diet/portion-control-guide.pdf.

[42] National Institutes of Health Serving Size Card. https://www.nhlbi.nih.gov/health/educational/wecan/downloads/servingcard7.pdf.

[43] Millen B.E., Morgan J.L. (1996). 2D Food Portion Visual.

[44] Barrington W.E., Beresford S.A., McGregor B.A., White E. (2014). Perceived Stress and Eating Behaviors by Sex, Obesity Status, and Stress Vulnerability: Findings from the Vitamins and Lifestyle (Vital) Study. J. Acad. Nutr. Diet..

[45] Littman A.J., White E., Satia J.A., Bowen D.J., Kristal A.R. (2006). Reliability and Validity of 2 Single-Item Measures of Psychosocial Stress. Epidemiology.

[46] Likert R. (1932). A Technique for the Measurement of Attitudes. Arch. Psychol..

[47] USDA MyPlate Dairy. https://www.myplate.gov/eat-healthy/dairy.

[48] Wahidiyat P.A., Yo E.C., Wildani M.M., Triatmono V.R., Yosia M. (2021). Cross-sectional Study on Knowledge, Attitude and Practice towards Thalassaemia among Indonesian Youth. BMJ Open.

[49] Okello G., Izudi J., Teguzirigwa S., Kakinda A., Van Hal G. (2020). Findings of a Cross-Sectional Survey on Knowledge, Attitudes, and Practices about COVID-19 in Uganda: Implications for Public Health Prevention and Control Measures. BioMed Res. Int..

[50] Basheer R., Bhargavi P.G., Prakash H.P. (2019). Knowledge, Attitude, and Practice of Printing Press Workers towards Noise-Induced Hearing Loss. Noise Health.

[51] Rozita W. (2006). Knowledge, Attitude and Practice (KAP) Survey on Dengue Fever in an Urban Malay Residential Area in Kuala Lumpur. Malays. J. Public. Health Med..

[52] Melki I.S., Beydoun H.A., Khogali M., Tamim H., Yunis K.A., National Collaborative Perinatal Neonatal N. (2004). Household Crowding Index: A Correlate of Socioeconomic Status and Inter-Pregnancy Spacing in an Urban Setting. J. Epidemiol. Community Health (1979).

[53] (2023). Eating Unhealthy Food. https://www.breastcancer.org/risk/risk-factors/eating-unhealthy-food#.

[54] Kotepui M. (2016). Diet and risk of breast cancer. Contemp. Oncol..

[55] Farvid M.S., Barnett J.B., Spence N.D. (2021). Fruit and Vegetable Consumption and Incident Breast Cancer: A Systematic Review and Meta-Analysis of Prospective Studies. Br. J. Cancer.

[56] Seiler A., Chen M.A., Brown R.L., Fagundes C.P. (2018). Obesity, Dietary Factors, Nutrition, and Breast Cancer Risk. Curr. Breast Cancer Rep..

[57] Sbaity E., Bejjany R., Kreidieh M., Temraz S., Shamseddine A. (2021). Overview in Breast Cancer Screening in Lebanon. Cancer Control.

[58] Lebanese Ministry of Public health National Cancer Plan. 2023–2028. https://www.moph.gov.lb/userfiles/files/About%20MOPH/StrategicPlans/National%20Cancer%20Plan%202023-2028/NATIONAL%20CANCER%20PLAN%20OFFICIAL%20VERSION.pdf.

[59] Al-Naggar R.A., Chen R. (2011). Nutrition and Cancer Prevention: Knowledge, Attitudes and Practices among Young Malaysians. Asian Pac. J. Cancer Prev..

[60] Olodu M., Adeomi A., Opia F., Otuyemi O., Ajayi B., Rasaq A. (2021). Nutrition and Cancer Prevention: An Assessment of Undergraduates’ Knowledge and Nutritional Practices. Niger. J. Health Sci..

[61] Jovanović G.K., Kresić G., Zezelj S.P., Mićović V., Nadarević V.S. (2011). Cancer and Cardiovascular Diseases Nutrition Knowledge and Dietary Intake of Medical Students. Coll. Antropol..

[62] American Institute for Cancer Research (2019). 2019 AICR Cancer Risk Awareness Survey. https://www.aicr.org/assets/can-prevent/docs/2019-Survey.pdf.

[63] Sanderson S.C., Waller J., Jarvis M.J., Humphries S.E., Wardle J. (2009). Awareness of Lifestyle Risk Factors for Cancer and Heart Disease among Adults in the UK. Patient Educ. Couns..

[64] Allen K. (2022). It’s Never Been More Important to Focus on Cancer Prevention. https://www.wcrf.org/its-never-been-more-important-to-focus-on-cancer-prevention/.

[65] Harnack L., Block G., Subar A., Lane S. (1998). Cancer Prevention-Related Nutrition Knowledge, Beliefs, and Attitudes of U.S. Adults: 1992 NHIS Cancer Epidemiology Supplement. J. Nutr. Educ..

[66] Hawkins N.A., Berkowitz Z., Peipins L.A. (2010). What does the public know about preventing cancer? Results from the Health Information National Trends Survey (HINTS). Health Educ. Behav..

[67] Lamore K., Ducrot P., Latino-Martel P., Soler M., Foucaud J. (2019). Diet, Physical Activity, Obesity, and Breastfeeding: How French People Perceive Factors Associated with Cancer Risk. Nutrients.

[68] Redeker C., Wardle J., Wilder D., Hiom S., Miles A. (2009). The launch of Cancer Research UK’s ‘Reduce the Risk’ campaign: Baseline measurements of public awareness of cancer risk factors in 2004. Eur. J. Cancer.

[69] Ryan A.M., Cushen S., Schellekens H., Bhuachalla E.N., Burns L., Kenny U., Power D.G. (2015). Poor Awareness of Risk Factors for Cancer in Irish Adults: Results of a Large Survey and Review of the Literature. Oncologist.

[70] Lagerlund M., Hvidberg L., Hajdarevic S., Fischer Pedersen A., Runesdotter S., Vedsted P., Tishelman C. (2015). Awareness of Risk Factors for Cancer: A Comparative Study of Sweden and Denmark. BMC Public. Health.

[71] World Cancer Research Fund (2018). Body Fatness and Weight. Gain and the Risk of Cancer. https://www.wcrf.org/wp-content/uploads/2021/01/Body-fatness-and-weight-gain_0.pdf.

[72] Sullivan H.W., Klassen A.C. (2007). Nutrition-related cancer prevention attitudes in low-income women. Prev. Med..

[73] Naja F., Nasreddine L., Awada S., El Sayed Ahmad R., Hwalla N. (2019). Nutrition in the Prevention of Breast Cancer: A Middle Eastern Perspective. Front. Public Health.

[74] Farvid M.S., Chen W.Y., Rosner B.A., Tamimi R.M., Willett W.C., Eliassen A.H. (2019). Fruit and Vegetable Consumption and Breast Cancer Incidence: Repeated Measures over 30 Years of Follow-Up. Int. J. Cancer.

[75] World Cancer Research Fund (2018). Wholegrains, Vegetables and Fruit and the Risk of Cancer. https://www.wcrf.org/wp-content/uploads/2020/12/Wholegrains-veg-and-fruit.pdf.

[76] Farvid M.S., Eliassen A.H., Cho E., Liao X., Chen W.Y., Willett W.C. (2016). Dietary Fiber Intake in Young Adults and Breast Cancer Risk. Pediatrics.

[77] Yang B., Ren X.L., Fu Y.Q., Gao J.L., Li D. (2014). Ratio of n-3/n-6 PUFAs and risk of breast cancer: A meta-analysis of 274135 adult females from 11 independent prospective studies. BMC Cancer.

[78] Fleet J.C., DeSmet M., Johnson R., Li Y. (2012). Vitamin D and Cancer: A Review of Molecular Mechanisms. Biochem. J..

[79] Cui Y., Rohan T.E. (2006). Vitamin D, Calcium, and Breast Cancer Risk: A Review. Cancer Epidemiol. Biomark. Prev..

[80] Arafat H.M., Omar J., Shafii N., Naser I.A., Al Laham N.A., Muhamad R., Al-Astani T.A.D., Shaqaliah A.J., Shamallakh O.M., Shamallakh K.M. (2023). The Association between Breast Cancer and Consumption of Dairy Products: A Systematic Review. Ann. Med..

[81] Guo J., Wei W., Zhan L. (2015). Red and Processed Meat Intake and Risk of Breast Cancer: A Meta-Analysis of Prospective Studies. Breast Cancer Res. Treat..

[82] Taha Z., Eltom S.E. (2018). The Role of Diet and Lifestyle in Women with Breast Cancer: An Update Review of Related Research in the Middle East. Biores Open Access.

[83] Ferguson L.R. (2010). Meat and Cancer. Meat Sci..

[84] Deliens T., Clarys P., De Bourdeaudhuij I., Deforche B. (2014). Determinants of Eating Behaviour in University Students: A Qualitative Study Using Focus Group Discussions. BMC Public Health.

[85] Hashim M., Abdelrahim D.N., Ahmed S., Tahir B., Youssef M., Mousannef J., Almazrouei M., Naja F., Al-Yateem N., Rahman S.A. (2022). Knowledge, Awareness, and Practices of University Students toward the Role of Dietary and Lifestyle Behaviors in Colorectal Cancer: A Cross-Sectional Study from Sharjah/Uae. Asian Pac. J. Cancer Prev..

[86] Yun T.C., Ahmad S.R., Quee D.K.S. (2018). Dietary Habits and Lifestyle Practices among University Students in Universiti Brunei Darussalam. Malays. J. Med. Sci..

[87] Naja F., Hwalla N., El Zouhbi A., Abbas N., Chamieh M.C., Nasreddine L., Jomaa L. (2020). Changes in Environmental Footprints Associated with Dietary Intake of Lebanese Adolescents between the Years 1997 and 2009. Sustainability.

[88] World Food Program Lebanon. https://www.wfp.org/countries/lebanon.

[89] Itani R., Mattar L., Kharroubi S., Bosqui T., Diab-El-Harake M., Jomaa L. (2022). Food insecurity and mental health of college students in Lebanon: A cross-sectional study. J. Nutr. Sci..

[90] Belogianni K., Ooms A., Lykou A., Moir H.J. (2022). Nutrition knowledge among university students in the UK: A cross-sectional study. Public. Health Nutr..

[91] Mousa T.Y., Dardas L.A. (2023). Nutrition knowledge, food security, and other risk factors in a sample of college students in Jordan: A cross-sectional design. Cogent Food Agric..

[92] U.S. Department of Health and Human Services The Teen Brain: 7 Things to Know. https://www.nimh.nih.gov/health/publications/the-teen-brain-7-things-to-know#:~:text=The%20brain%20finishes%20developing%20and,prioritizing%2C%20and%20making%20good%20decisions..

[93] Hearty Á.P., McCarthy S.N., Kearney J.M., Gibney M.J. (2007). Relationship between attitudes towards healthy eating and dietary behaviour, lifestyle and demographic factors in a representative sample of Irish adults. Appetite.

[94] Platikanova M., Yordanova A., Hristova P. (2022). Dependence of Body Mass Index on Some Dietary Habits: An Application of Classification and Regression Tree. Iran. J. Public. Health.

